# Effect of a daily outdoor access on the urination and defecation behaviors and nitrogen excretion by lactating cows

**DOI:** 10.3389/fvets.2025.1429638

**Published:** 2025-03-03

**Authors:** Lucia Bailoni, Sheyla Arango, Nadia Guzzo, Sarah Currò, Emanuele Bianco, Emilio Simonetti, Elena Zerbinati, Simona Rainis, Cristina Sartori

**Affiliations:** ^1^Department of Comparative Biomedicine and Food Science (BCA), University of Padova, Legnaro, Italy; ^2^Regional Agency for the Rural Development (ERSA), Pozzuolo del Friuli, Italy; ^3^Department of Agronomy Food Natural Resources Animals and Environment (DAFNAE), University of Padova, Legnaro, Italy

**Keywords:** welfare, dairy cattle, animal management, external paddock, daily exit

## Abstract

This study aimed to assess the urination and defecation frequency along with the nitrogen excretion produced by lactating cows spending either 2 or 4 h a day in an outdoor exercise area, to then estimate the load of cows allowed considering the nitrogen limitation in manure established by the EU directive. Six Italian Simmental lactating cows housed in a free-stall were paired and alternatively subjected to the following exit managements: no daily outdoor access, a 2-h daily outdoor access (U2; from 11:30 a.m. to 1:30 p.m.), and a 4-h daily outdoor access (U4) divided into a morning (U4a; 9:00 to 11:00 a.m.) and an afternoon (U4b; 2:00 to 4:00 p.m.) exit. Using a crossover design, each pair of cows was subjected to each exit management for a period of 2 weeks, then switched twice, until the completion of 6 weeks of evaluation in order to ensure all the three different group combinations. The study considered as treatments the two exit managements: U2 and U4. Cows in the paddock urinated and defecated on average 0.76 and 0.94 times per hour, respectively. The content of nitrogen in urine and feces was 0.42% and 1.96% on average, respectively. Excretion variables such as nitrogen output of urine, nitrogen output of feces and total nitrogen output were 6.19, 4.91, and 11.10 g/h on average respectively, and they were not affected by the treatment. The total nitrogen output by the cows spending 2 or 4 h of daily outdoor activity was 22.68 and 44.07 g per cow, respectively (*P* < 0.001). In conclusion, the duration of the outdoor exit did not influence the number of urination or defecations events per hour, the nitrogen excretion output in urine and feces, and therefore, the total nitrogen output per hour in the outside paddock. Considering a 2 or 4-h daily exit along the whole year and the limit of nitrogen of 170 kg N/ha/year given by the EU Directive, the maximum stocking rate per hectare would be equal to 21 and 11 dairy cows, respectively.

## 1 Introduction

The global rise in milk demand necessitates an increase in the feed efficiency of high-producing dairy cows that require larger amounts of energy and protein in feed. Consequently, this greatly increases the need for cultivated areas, such as corn and soybean fields, at the expense of grazing lands (1). In many countries worldwide, dairy farming intensification has led to increased indoor confinement of animals (2). However, such indoor housing management can result in various detrimental consequences, including a surge in health issues, decreased expression of natural behaviors, and diminished fitness, potentially leading to shorter lifespans (3). Consequently, alternative approaches to confined farming management have been proposed, aiming to grant animals access to external areas that facilitate movement opportunities for a suitable duration (4).

A good housing is considered a welfare principle for dairy cows, in which the ease of movement could be guaranteed by giving access to an outdoor loafing area (5). Exercise plays a vital role in maintaining the fitness of legs and muscles and in averting abnormal behaviors during periods of lying down or standing up, which could heighten the risk of traumatic udder injuries (6). Studies of Somers et al. (7) and Becker et al. (6) established a direct correlation between cows' health and outdoor movement. Gustafson (8) emphasized the positive impact of outdoor accessibility, particularly for tied dairy cows with limited social interaction. Outdoor exercise in a paddock improves the expression of natural behaviors, such as socialization and environmental exploration (9, 10) and diminishes the occurrence of hock lesions (11). Smid et al. (12) also observed positive oestrus behaviors when cows experienced outdoor access. Additionally, studies by Hernandez-Mendo et al. (13) and McLellan et al. (14) highlighted the potential for lame cows to enhance their gait in a relatively short time on pasture, indicating the positive effects of comfortable surfaces in aiding recovery from hoof and leg injuries.

Numerous factors, including indoor housing systems (tie-stall or free-stall), outdoor area characteristics (size, floor type, presence of trees or shaded surfaces), type of outdoor access (farmer-controlled or free), milking systems (manual or automatic), and duration and frequency of paddock access, influence the benefits derived from external access for cows as reported by Shepley et al. (4, 15).

Conversely, a crucial aspect to consider in this type of dairy management is the significant nitrogen excretion by cows during grazing or outdoor stays, contributing significantly to atmospheric N-NO3- release and soil/water pollution (16–18). Studies by Oudshoorn et al. (19), White et al. (20), and Carpinelli et al. (21) have explored strategies to mitigate nitrogen accumulation while grazing. Hirata et al. (22) investigated the diurnal excretion patterns of grazing cattle and the potential use of defecation frequency as an estimator of fecal nutrient deposition and accumulation on pasture. Additionally, Marshall et al. (23) proposed a urine sensor as a tool to study urination behavior and estimate urine volume per urination event in grazing dairy cows. In that way, we can use defecation and urination events to assess the nitrogen excretion released into the soil.

Despite the increasing interest for providing cows an outdoor access to improve welfare, a limited number of studies exist regarding the measurements of dairy cows' total nitrogen excretion during fixed-time stays in external areas without access to pasture. The present study aimed to assess urination and defecation frequency along with the nitrogen excretion produced by Simmental lactating cows that spent 2 or 4 h a day in an outdoor exercise area, to also provide an approach for the estimation of the stocking rate to use in this external area considering the nitrogen limitation (170 kg N/ha/year) in manure established by the EU directive.

## 2 Materials and methods

### 2.1 Animals and experimental design

The trial was carried out at the dairy farm of the Institute of Higher Education (ISIS Paolino d'Aquileia) located in Cividale del Friuli (Udine, Italy). The experimental location was a free stall equipped with an automatic milking system (Lely Astronaut 2, Lely, Maassluis, Netherlands). All experimental procedures were approved by the ethical committee at the University of Padova (approval number 36/2023) and carried out according to the directive 2010/63/UE of the European Parliament on the protection of animals used for scientific purposes and the Italian law on animal care (Legislative Decree No. 26 of 14 March 2014) (36).

Study subjects were cows of the breed named “Pezzata Rossa Italiana.” This Italian breed, born in 1986, has a National Breed Association (ANAPRI) and an Official Breed Pedigree Book. The breeds' name is also mentioned in several scientific publications as Italian Simmental cows. Six Simmental lactating cows were chosen for the study based on parity (2.0 ± 1.4), days in milk (103 ± 35), and absence of mastitis and lameness. The sample size was chosen following the literature (24). Cows were divided into three pairs, which were alternatively assigned to the following exit managements: (i) no daily outdoor exit (CTR); (ii) a 2-h daily exit (U2); (iii) a 4-h daily exit (U4). The CTR group stayed the whole-day inside the free-stall, the U2 cows had a midday daily outdoor access (from 11:30 a.m. to 1:30 p.m.), and the U4 cows had firstly a morning outdoor access (U4a: from 9:00 to 11:00 a.m.) and then an afternoon outdoor access (U4b: from 2:00 to 4:00 p.m.). This research is part of a broader experimental setting developed from a Latin square design aimed at sampling parameters inside the stall, either once or repeatedly during three experimental periods. However, the present work focused exclusively on variables collected outdoors, considering as treatments the two exit managements (U2 and U4) with no sampling performed from the CTR group. This allowed to capture the difference between moving animals outdoors once a day or twice a day, which is twice as long. In addition, to distinguish between the timing of outdoor access, morning (U4a) and midday (U2) exits were compared separately using the same model and reported as a Supplementary material. The afternoon timing (U4b) was not considered to avoid repetitive measurements within the same animal and also because this pair of cows had already exited in the morning.

The experiment took place from October 31 and December 7, 2022, during the winter season. Three weeks before the experiment was considered as an adjustment period for cows to get used to the paddock. No data was recorded during the adjustment period, due to its aim. The experimental trial had a duration of 6 weeks divided into three subsequent periods of 2 weeks. Using a crossover design, each pair of cows was subjected to each treatment for 2 weeks, then switched, until the completion of 6 weeks of evaluation in order to ensure all the three different group combinations. An overview of the experimental setting is included as a Supplementary Table S1. The animals did not have prior experience with outdoor exercise and they were not forcibly driven, they moved voluntarily until they reached and stayed freely in the outdoor paddock.

### 2.2 Diet

All animals were fed a total mixed ration (TMR) based on grass-silage and alfalfa hay (Table 1) and maintained a forage:concentrate ratio of 62:38 to fulfill energy requirements of lactating dairy cows, following the guidelines outlined by the National Research Council (25). The TMR was distributed once a day during the morning (around 8.30 am). TMR samples were collected at the beginning and the end of the experiment, and then analyzed using near-infrared spectroscopy (NIRs). Additional compound feed was available depending on daily milk yield during the access to the automatic milking system of the farm. Fresh water was available ad libitum, both inside and in the outdoor area.

**Table 1:** Ingredients and chemical composition of the total mixed ration^a^.

**Ingredients, % DM**	
Grass silage	26.4
Alfalfa hay	32.8
Barley straw	2.7
Corn meal	17.3
Barley meal	12.4
Compound feed^b^	8.4
**Chemical composition, % DM**	
DM, % as fed	51.2
Crude Protein	14.7
Ash	8.6
Lipids	2.0
NDF	38.7
ADF	24.7
Lignin	3.8
Starch	21.1
NE_L_, MJ/kg of DM^c^	5.80

a TMR = Total mixed ration provided by the automatic feeder. The amount of the compound feed available during milking is excluded.

b Chemical composition of compound feed: Moisture 13.00%, Crude Protein 18.50%, Lipids 3.20%, Crude Fiber 6.10%, Sodium 0.43%, Ash 7.40%.

c According to NRC (25).

### 2.3 Outdoor area

The outdoor area, encompassing 670 m^2^ and surrounded by an electric fence, adhered to the guidelines established by the Royal Society for the Prevention of Cruelty to Animals (26). This paddock was used just as an exercise area for the animals. Before the experimental period, the paddock grass was trimmed to prevent grazing. Inside and close to the outdoor area, there were four trees of a few meters height that provided shadow in some moments of the day. Animals were milked before accessing this external area with the objective of ensuring that they did not accumulate excessive quantities of milk before exiting. In fact, with the automatic milking system, not all cows spontaneously choose to be milked before the time to go outside.

### 2.4 Behavioral observations and urine and feces collection

They involved 12 trained observers working in pairs chosen from students of the institute. Cows of U2 and U4 were allowed to exit 5 days per week, from Monday to Friday, during each period (2 weeks). Only Mondays and Thursdays were considered for evaluation, meaning 4 days per period. Two of these days were lost due to adverse weather conditions. Frequency of behaviors were evaluated during the hours in which the pair of cows were inside the paddock. Whenever the animals defecated or urinated, the observers recorded the identification of the animal and the occurrence. The dataset included a total of 40 observations.

A direct technique that implies the direct measurement of the urine flow and the fecal deposition was used and done by a trained student of the Institute. As soon as a cow exhibited a urination behavior (body slightly bent back, tail lifted, back considerably arched, and hind legs placed forward and apart), urine was collected in a bucket using a long stick without startling the animal and weighed. Samples of urine (15 mL) were collected and handled following the procedure of Knowlton et al. (27) until analyzed. Fresh feces were immediately collected from the ground using a shovel right after each defecation, special care was taken to ensure that no soil or grass was picked up and weighed. Samples of feces (150 ± 17.56 g) were collected, and pre-dried at 60°C for 48 h, then the residual moisture was determined after drying at 103°C. Total nitrogen content in urine and pre-dried feces were analyzed using the Kjeldahl method following the AOAC (28) standard procedures.

### 2.5 Statistical analysis

After preliminary analyses aimed to find out the best statistics for running the data, including both linear models and mixed models, all parameters (behavioral and excretion) were evaluated using a mixed model analysis (MIXED procedure, SAS Institute Inc., Cary NC, 2014):


$$\begin{array}{l} {\text{Y}}_{\text{ijklmn}}=\text{μ}+{\text{T}}_{\text{i}}+{\text{P}}_{\text{j}}+{\text{G}}_{\text{k}}+\text{D}:{\text{P}}_{\text{jl}}+\text{C}:{\text{G}}_{\text{km}}+{\text{e}}_{\text{ijklmn}} \end{array}$$

where, Y_ijklmn_ is the target individual parameter, μ is the overall mean, T is the fixed effect of the treatment, that is the outdoor management (two levels: U2 and U4); P is the fixed effect of the experimental period, with three levels; G is the effect of the group, that is the specific cow pair, maintained over the whole trial (three levels); D is the fixed effect of the day of observation within period, representing the repetition of the sampling, C is the random effect of the cow within group (six levels), and e_ijklmn_ is the residual error (SAS Institute Inc. Cary NC, 2014). Least square means were computed on the levels of T effect and compared using a Student *t*-test. As the treatments in comparison have two different durations, 2 and 4 h, some of the variables were also calculated per hour in order to make comparisons within the same time interval. The behavioral variables were frequency of urination and defecation as absolute number of events (n) and hour occurrence (n/h). The excretion parameters were: fresh urine weight (kg), N content in urine (%), N output of urine (g), N output of urine per hour (g/h), fresh fecal weight (kg), dry matter of feces (%), N content of feces (%DM), N output of feces (g), N output of feces per hour (g/h), total N output (g) and total N output per hour (g/h). In addition, a supplementary model was run on a subset excluding the U4 observations to compare two timings as treatment: morning (U4a) and midday (U2) (Supplementary Table S2). These two timings were chosen because they had a duration of 2 h and were the first exits of the animals (U4b was the second exit of the day). The same variables, except for the ones expressed on hourly basis, were accounted for. In the analysis we considered *P* < 0.05 to determine statistical significance.

Finally, the possible associations among the variables considered in the study, in particular expressed either as total or per hour, were investigated through Pearson correlations. The statistical significance of correlations was stated through a pairwise Student *t*-test (CORR procedure, SAS Institute Inc., Cary NC, 2014), and a Bonferroni correction for multiple comparison was then applied for each group of variables.

## 3 Results

### 3.1 Urination and defecation behaviors

Table 2 reports the descriptive statistics of the variables. During the time spent in the external paddock, lactating dairy cows urinated and defecated on average 0.76 and 0.94 times per hour, respectively. Urination and defecation frequencies in lactating cows that were allowed to spend time in an outside paddock varied from 0 to 1.75 and from 0 to 2 events per hour, respectively.

**Table 2:** Descriptive statistics on the variables considered in the study for the two outdoor access treatments.

	**Mean**	**SD**	**Min**	**Median**	**Max**
**Frequency**					
Urination (n tot)	2.36	1.64	0	2.00	7.00
Defecation (n tot)	2.83	1.63	0	2.00	7.00
Urination (n/h)	0.76	0.40	0	0.63	1.75
Defecation (n/h)	0.94	0.42	0	1.00	2.00
**Excretion**					
Urine weight (kg)	4.15	3.65	0	3.79	20.65
N content of urine (%)	0.42	0.23	0	0.45	0.86
N output of urine (g)	18.38	13.29	0	16.64	50.80
N output of urine (g/h)	6.19	4.13	0	4.82	15.41
Fecal weight (kg)	4.24	2.49	0	3.41	9.74
Dry matter of feces (%)	0.71	0.04	0	0.62	0.29
N content of feces (%DM)	1.96	0.48	0	2.03	2.68
N output of feces (g)	14.74	8.34	0	11.88	36.60
N output in feces (g/h)	4.91	2.23	0	4.92	10.52
Total N output (g)	33.12	15.96	7.66	32.30	74.49
Total N output (g/h)	11.10	4.17	3.42	10.61	21.49

The ANOVA on the fixed effects of the model (Table 3) showed a significant effect of the treatment (*P* < 0.001) and period (*P* < 0.05) for the frequency of both behaviors expressed as total of number of events, but not when expressed per hour (*P* > 0.05). There were not significant effects of the group of cows, within animals, on the number of urinations and defecations per hour.

**Table 3:** ANOVA (*F*-value) on the fixed effects of the urination and defecation activities and their nitrogen excretion.

	**Treatment *(T)***	**Period *(P)***	**Group *(G)***	**Day within *P***
**Frequency**				
Urination (n tot)	19.5745^***^	4.9636^*^	1.2695	0.6009
Defecation (n tot)	30.3807^***^	5.6805^**^	5.6889	1.0502
Urination (n/h)	0.1611	4.2490^*^	0.7015	0.3970
Defecation (n/h)	0.0191	2.9771	3.1392	1.0109
**Excretion**				
Urine weight (kg)	8.3655^**^	1.7239	5.2919	1.0134
N content in urine (%)	0.0109	1.0615	5.0010	3.0551^*^
N output of urine (g)	8.7991^**^	0.2485	0.0133	1.6045
N output of urine (g/h)	0.0467	0.3651	0.1164	1.5904
Fecal weight (kg)	43.1297^***^	8.4790^*^	2.0504	1.8188
Dry matter of feces (%)	0.5373	0.9540	1.4387	1.5786
N content in feces (%DM)	0.4487	2.3292	2.4021	0.6092
N output in feces (g)	29.2261^***^	5.4222^*^	0.1389	1.4309
N output in feces (g/h)	0.0044	7.1778^**^	0.4584	1.6940
Total N output (g)	32.6525^***^	0.4148	0.0873	1.6289
Total N output (g/h)	0.0591	0.3373	0.0045^**^	1.6295

^***^P < 0.001, ^**^P < 0.01, ^*^P < 0.05.

### 3.2 Nitrogen excretion

The ANOVA on the fixed effects of the model (Table 3) showed a significant effect of the treatment (*P* < 0.01) for almost all the variables, but not for the ones expressed per hour. When cows stay outside 2 or 4 h daily, they did not show significant differences (*P* > 0.05) in the percentage of nitrogen in urine, the percentage of nitrogen in feces or the dry matter of feces.

The quantity of feces produced during the 2 and 4 h spent outside was significantly different (2.74 vs. 5.99 kg; *P* < 0.001) because of the duration of the stay (Table 4). But also, when comparing intervals of 2 h, an effect of the exit time was found between morning (U4a) and midday (U2) showing a statistical difference of the fecal weight produced (3.82 vs. 2.74 kg; *P* < 0.05) (Supplementary Table S3).

**Table 4:** Least square means of the urination and defecation activities and their nitrogen excretion for the two outdoor access treatments.

	**U2**	**U4**	**SE**	***P***
**Frequency (n)**				
Urination (n tot)	1.46	3.10	0.39	<0.001
Defecation (n tot)	1.86	3.79	0.25	<0.001
Urination (n/h)	0.73	0.77	0.13	ns
Defecation (n/h)	0.93	0.95	0.09	ns
**Excretion**				
Urine weight (kg)	2.78	5.50	0.67	<0.001
N content of urine (%)	0.42	0.41	0.04	ns
N output of urine (g)	12.50	23.86	2.71	0.006
N output of urine (g/h)	6.25	5.96	0.93	ns
Fecal weight (kg)	2.74	5.99	0.35	<0.001
Dry matter of feces (%)	17.51	16.60	1.06	ns
N content of feces (%DM)	2.03	1.93	0.11	ns
N output of feces (g)	10.19	20.22	1.31	<0.001
N output in feces (g/h)	5.09	5.05	0.94	ns
Total N output (g)	22.68	44.07	2.65	<0.001
Total N output (g/h)	11.34	11.02	0.94	ns

U2 (2 hour outdoor access a day: from 11:30 a.m. to 1:30 p.m.); U4 (4 hour outdoor access a day: from 9:00 to 11:00 a.m. and from 2:00 to 4:00 p.m.). SE, Standard Error.

As expected, an evident difference due to the duration of the outdoor exit was found in variables such as the N output of urine (g), N output in feces (g) and total N output (g) when cows spent 2 and 4 h in the outside paddock. The effect of the group and the effect of the day of observation within period were only significant for the total nitrogen output per hour (*P* < 0.01) and for the percentage of nitrogen content in urine (*P* < 0.05) respectively. The percentage of nitrogen content in urine and feces was 0.42 ± 0.23 % and 1.96 ± 0.48 % respectively, and the total nitrogen output per hour was 11.10 ± 4.17 g (Table 2).

### 3.3 Correlations among variables

Tables 5, 6 report the Pearson (r) correlations of some selected variables. Frequencies of urination (n tot) and defecation (n not) were positively associated with their respective amount of urine (kg) (*r* = 0.48; *P* < 0.0071) and feces (kg) (*r* = 0.66; *P* < 0.0014) produced by cows (Table 5). Neither the number of urinations nor the number of defecations produced per hour in the outside paddock was significantly correlated with the total nitrogen output per hour of lactating cows (Table 6). High and significant correlations were found between urine weight (kg) and the N output in urine expressed in grams (*r* = 0.67; *P* < 0.0014). The same with fecal weight (kg), that was highly and significantly correlated with the N output in feces expressed in grams (*r* = 0.95; *P* < 0.0014) (Table 5). Differently from urination, the behavior variable of frequency of defecation (n tot) was more positively correlated (*r* = 0.56; *P* < 0.0014) with its excretion parameter of N output of feces (g). The total nitrogen output (g) was significantly highly correlated to N output of urine (g) (*r* = 0.85; *P* < 0.0014) and also to N output of feces (g) (*r* = 0.56; *P* < 0.0014).

**Table 5:** Pearson correlations among variables expressed as total (*n* = 40).

**Variable**	**1**	**2**	**3**	**4**	**5**	**6**	**7**
1. Urination (n tot)	1	0.57^**^	0.48^*^	0.35	0.25	0.09	0.34
2. Defecation (n tot)		1	0.44^*^	0.15	0.66^**^	0.56^**^	0.42^*^
3. Urine weight (kg)			1	0.67^**^	0.34	0.16	0.64^**^
4. N output of urine (g)				1	0.13	0.04	0.85^**^
5. Fecal weight (kg)					1	0.95^**^	0.60^**^
6. N output of feces (g)						1	0.56^**^
7. Total N output (g)							1

^*^P < 0.0071, ^**^P < 0.0014, calculated using Bonferroni correction.

**Table 6:** Pearson correlations among variables expressed per hour (*n* = 40).

**Variable**	**1**	**2**	**3**	**4**	**5**
1. Urination (n/h)	1	0.17	0.04	−0.33	−0.14
2. Defecation (n/h)		1	−0.03	0.38^*^	0.18
3. N output of urine (g/h)			1	−0.25	0.86^**^
4. N output in feces (g/h)				1	0.28
5. Total N output (g/h)					1

^*^P < 0.01, ^**^P < 0.002, calculated using Bonferroni correction.

## 4 Discussion

### 4.1 Urination and defecation activities

In this experiment, the frequency of urination and defecation by lactating cows were primarily studied during an outdoor stay of 2 and 4 h in a paddock. There is no data on the average daily urination frequency in cows when they stay or just spend some time in an outside paddock so data from studies of grazing cows was considered. Lantinga et al. (29) reported a tendency for cows to urinate about 12 times per day during grazing. Oudshoorn et al. (19) reported averages of urination and defecation frequencies of 6.5 and 10.5 times per day, respectively, unaffected by three different grazing times. More recently, Ravera et al. (30) studied the differences in urination frequency of non-lactating dairy cows subjected to two different feeding systems based on the access to two different crops (kale vs. fodder beet) and found a frequency of urination that ranged from 8.2 to 12.3 times per day. Higher values, from 13.3 to 17 times per day, were observed by Nguyen et al. (31), who tested plantain (*Plantago lanceolate L*.), perennial ryegrass (*Lolium perenne L*.) and white clover (*Trifolium repens L*.) as forages in a pastoral system. Moreover, Hirata et al. (22) described the daily pattern (24 h) of excretion in grazing animals using Pensacola bahiagrass (*Paspalum notatum*) evaluated over 2 years and stated that the daily frequency of urination of cows was significantly higher in summer than in autumn (8.0 vs. 5.9 times/day). The highest frequency of urination activity, considering only the autumn season, occurred between 8:00-9:00 a.m. and 12:00 a.m.-1:00 p.m. According to our results, the frequency of urination of the animals when they were outside was 0.76 times per hour. Assuming this value as the average for diurnal production over a period of 12 h, the number of times for urination would be estimated as 9.12 times per day that fits into the previously mentioned studies. Evidently, the total frequency of urination was higher when cows spent 4 h outside rather than two because of the duration of the stay in the paddock. But, the results showed no differences for the urination frequency expressed per hour.

Currently, information regarding cow defecation events raised in a stall but with different exit managements is limited due to the novelty of these types of approaches. However, studies from grazing cows were considered for the discussion of the results. The frequency of defecation, averaging 0.94 times per hour spent outdoors, was also unaffected by the treatment. And again, if we consider a diurnal production over a period of 12 h, the number of times for defecation would be estimated as 11.28 per day which agrees with Lantinga et al. (29) that reported a tendency for cows to defecate about 12 times per day during grazing.

### 4.2 Nitrogen from urinary and fecal output

Under the conditions of this study, the results of this experiment identified significant differences in defecation and urination excretion variables when cows exited once or twice a day mostly due to the duration of the stay. Spending four rather than 2 h in the outside paddock means twice of the time for urinating and defecating. As already discussed above, the 4-h daily exit resulted in more urination and defecation events which directly affected the quantity of urine and feces produced. Therefore, the nitrogen output of both behaviors resulted in a higher total nitrogen output for the U4 treatment.

#### 4.2.1 Urination

The amount of urine each cow produced in the present study was 1.38 kg per hour spent in the external paddock. Assuming this value as the average for diurnal production, over a period of 12 h the total amount of urine would be estimated as 16.56 kg/day. This result is comparable to or lower than the total urine volume of 17.97 and 29.91 L/d from non-lactating dairy cows in a crop wintering system based on kale or fodder beet, respectively, obtained by Ravera et al. (30).

Even though the literature expressed that urine nitrogen concentration varies with many factors, the percentage of nitrogen in urine (0.42 ± 0.23 on average) was not affected by the duration of the daily exit of the cows. The effect of the diet on cow urine nitrogen concentration was not a concern in this study as all the animals received the same diet meaning the same protein content. It is noteworthy that this result is much lower than the mean of 4.63% reported by Nguyen et al. (31) for lactating cows while grazing plantain-based pastures. This study observed variations in urine nitrogen concentration between days within period, but not between the individual cows.

#### 4.2.2 Defecation

As the U4 group spent twice longer in the outside paddock than the U2 group, it was normal to find the double production of feces and therefore twice nitrogen output in feces expressed in grams. This was also supported by the fact that there were no differences between the percentage of dry matter in feces and the nitrogen concentration in feces (%DM) in the two treatments. Nevertheless, nitrogen output in feces expressed as grams per hour was similar between the two exit times managements.

A study in grazing dairy cows reported that fecal weight had a non-uniform distribution along the day, with a tendency of higher amounts during the night than on daytime (22). This is why this study tried to analyze deeper the effect of timing by just considering and comparing intervals of the same duration (2 h), and an interesting finding regarding the fecal weight was found (Supplementary Table S2). Cows that exited 2 h during the morning (U4a) produced more feces than the ones exiting at midday (U2).

#### 4.2.3 Nitrate directive and stocking rate estimation

Space requirements, especially as herd sizes increase, is one of the main reasons why this type of outdoor access may or may not be provided to dairy cows by the farmers. To estimate this outdoor space, the total nitrogen excretion is required in order to not overload the land with nitrogen. In this study, the total nitrogen output by the cows spending 2 or 4 h of daily outdoor activity was 22.68, and 44.07 g per cow, respectively (*P* < 0.001). As the time spent for U4 was twice longer than U2, this value was found to be twice higher. It is desirable to reduce the risk of nitrogen leaching in a farm management. Furthermore, the European Union Nitrates Directive 91/676/EEC (32) has the objective to decrease nitrate leaching and one of the measures for the vulnerable zones is a maximum application standard of manure of 170 kg N/ha/year. To comply with this regulation and if we consider the actual diet and an everyday access to the external paddock throughout the whole year, the maximum stocking rate per hectare would be equal to 21 and 11 dairy cows for the 2- and 4-h daily exit, respectively. Noteworthy is the fact that assuming the abovementioned standard of manure as suitable for all of Europe may be overly simplistic, requiring country-specific considerations (33).

#### 4.2.4 Correlations

The frequencies of urination and defecation (n/h) were not correlated to each other, which may reflect that when cows spend time in an outside paddock they urinate or defecate indistinctly and not together or within a short period. The significant medium correlation between the behavioral variable defecation frequency and the excretion variable nitrogen output of feces (g/h), suggest the possibility to use the behavioral monitoring as a good proxy for estimating the nitrogen impact of feces into the soil. Usually, nitrogen output is estimated using intake and the chemical composition of the diet (34, 35). Whereas, literature is lacking in studies on the relationship between defecation behavior and nitrogen content in feces, but the recent possibility of using the latest sensor technology for massively acquiring behavioral information may suggest a possible application of this kind of data for estimating the nitrogen impact in the soil.

As total nitrogen output was found to correlate more strongly with urine than with feces, values could suggest that the quantity of urine excreted, along with its nitrogen content, could serve as good predictors for total nitrogen output in lactating cows.

## 5 Conclusions

In conclusion, a 2- or 4-h daily exit in lactating dairy cows had no influence on the urination and defecation frequency per hour in the outside paddock. Total nitrogen output per hour was unaffected by the treatment as a consequence of similar nitrogen excretion outputs per hour in urine and feces. The 2-h daily exit will allow a higher stocking rate of cows that will comply with the EU maximum application standard of manure for vulnerable zones. Due to the differences found in fecal weight between morning and midday, different timings of the day could be further investigated in order to choose the best moment of the day for the outdoor exit with a minimum environmental nitrogen impact. The findings from this study may be additionally validated by additional research investigating the impact of outdoor exits also in terms of production and animal wellbeing.

## Data Availability

The original contributions presented in the study are included in the article/Supplementary material, further inquiries can be directed to the corresponding author.
