# Supplementary material for: Effect of a daily outdoor access on the urination and defecation behaviors and nitrogen excretion by lactating cows
Source: Front Vet Sci. 2025 Mar 3;12:1429638. doi: 10.3389/fvets.2025.1429638 (PMC11913454; doi:10.3389/fvets.2025.1429638)
Supplement: Supplementary file 1 [file Table_1.docx]

Supplementary Table S1. Experimental design used in the study

| Experimental Period | Day of sampling | Treatments | | N° Records |
| --- | --- | --- | --- | --- |
|  |  | U2 | U4 |  |
|  | 1 | Cow1; Cow2^1^ | Cow3; Cow4^2^ | *4* |
| 1 | 2 | Cow1; Cow2 | Cow3; Cow4 | *4* |
|  | 3 | Cow1; Cow2 | Cow3; Cow4 | *4* |
|  | 4 | No sampling | |  |
|  | 1 | Cow3; Cow4 | Cow5; Cow6^3^ | *4* |
| 2 | 2 | Cow3; Cow4 | Cow5; Cow6 | *4* |
|  | 3 | Cow3; Cow4 | Cow5; Cow6 | *4* |
|  | 4 | Cow3; Cow4 | Cow5; Cow6 | *4* |
|  | 1 | Cow5; Cow6 | Cow1; Cow2 | *4* |
| 3 | 2 | Cow5; Cow6 | Cow1; Cow2 | *4* |
|  | 3 | No sampling | |  |
|  | 4 | Cow5; Cow6 | Cow1; Cow2 | *4* |
| *N°. records* | | *20* | *20* | *40* |

^1^ Cow1; Cow2 = Group 1; ^2^ Cow3; Cow4 = Group 2; ^3^ Cow5; Cow6 = Group 3.

Supplementary Table S2. ANOVA (F-value) on the fixed effects of the urination and defecation activities and their nitrogen excretion during the two timings: morning (U4a) and midday (U2) of outdoor access.

|  | Timing (T) | Period (P) | Group (G) | Day within P |
| --- | --- | --- | --- | --- |
| Frequency |  |  |  |  |
| Urination (n) | 0.2384 | 2.8046 | 0.3772 | 0.6789 |
| Defecation (n) | 0.8277 | 1.9637 | 2.0951 | 1.3367 |
| Excretion |  |  |  |  |
| Urine weight (kg) | 0.0031 | 0.2091 | 3.2983 | 0.6788 |
| N content in urine (%) | 0.8358 | 0.6706 | 2.6937 | 2.3933 |
| N output of urine (g) | 0.4973 | 0.4977 | 0.2782 | 1.3459 |
| Faecal weight (kg) | 5.3869* | 6.0075 | 0.5878 | 1.3607 |
| Dry matter of faeces (%) | 1.1600 | 1.3794 | 1.2123 | 1.5464 |
| N content in faeces (%DM) | 1.1100 | 4.0616* | 0.9801 | 0.6036 |
| N output in faeces (g) | 3.4712 | 5.9956** | 0.0321 | 1.1588 |
| Total N output (g) | 2.4730 | 0.6533 | 0.3328 | 1.4615 |

****P* < 0.001, ***P* < 0.01, **P* < 0.05.

Supplementary Table S3. Least square means of the urination and defecation activities and their nitrogen excretion during the two timings: morning (U4a) and midday (U2) of outdoor access.

|  | Morning  (U4a) | Midday (U2) | SE | *P* |
| --- | --- | --- | --- | --- |
| Frequency (n) |  |  |  |  |
| Urination | 1.57 | 1.46 | 0.25 | ns |
| Defecation | 2.10 | 1.86 | 0.18 | ns |
| Excretion |  |  |  |  |
| Urine weight (kg) | 2.81 | 2.78 | 0.45 | ns |
| N content of urine (%) | 0.49 | 0.42 | 0.05 | ns |
| N output of urine (g) | 14.79 | 12.50 | 2.30 | ns |
| Faecal weight (kg) | 3.82 | 2.74 | 0.33 | 0.029 |
| Dry matter of faeces (%) | 16.18 | 17.51 | 1.11 | ns |
| N content of faeces (%DM) | 2.16 | 2.03 | 0.11 | ns |
| N output of faeces (g) | 13.15 | 10.19 | 1.35 | ns |
| Total N output (g) | 27.94 | 22.68 | 2.47 | ns |

^1^Timing of the two-hour outdoor exits U4a (from 11:30 a.m. to 1:30 p.m) and U2 (from 9:00 to 11:00 a.m.)
